# Autonomous dynamic economic dispatch with limited fuel and renewable energy sources using marine predators optimizer

**DOI:** 10.1038/s41598-026-48247-2

**Published:** 2026-04-27

**Authors:** Mahmoud Ibrahim Mohamed, Alaa F. M. Ali, Ali M. Yousef, Ahmed A. Hafez

**Affiliations:** Electrical Engineering Department, Assiut University, Assiut, Egypt

**Keywords:** Dynamic economic emission dispatch, Dynamic generating capacity, Fuel shortages, Walrus optimization algorithm, Marine predators algorithm, Energy science and technology, Engineering

## Abstract

This article proposes a simple and reliable Dynamic Economic Emission Dispatch (DEED) model for a power system integrating Renewable Energy Sources (RES), including photovoltaic (PV) systems and wind energy. The thermal units in the system under concern are operating under limited fuel constraints. The proposed DEED effectively manages limited fuel conditions through Dynamic Generation Capacity (DGC). The Walrus Optimization Algorithm (WaOA) and Marine Predators Algorithm (MPA) are competing for the implementation of the proposed DEED with DGC and RES. The performance of the competing optimizers is compared against a mature meta-heuristic optimizer, Particle Swarm Optimization (PSO). The MPA is the most promising candidate, significantly outperforming the contenders, WaOA and PSO. For the 1000 MW load scenario, MPA and WaOA achieved a reduction in generation cost compared to PSO. Moreover, MPA substantially reduced the variability of results, demonstrating superior consistency compared to the competing optimizers. The proposed energy management strategies are tested on a system with 10 units under various operating scenarios. The results showed that the RES for power systems with limited/shortage fuel scenarios were insufficient to meet the required load, as fuel shortages tend to occur suddenly and unpredictably, thereby reducing system security. The DEED approach, incorporating both DGC and RES, achieved a notable reduction in fuel costs and emissions compared to the full fuel scenario and the fuel shortage scenario without RES. The results highlight the potential of integrating both DGC and RES into the DEED framework, demonstrating effectiveness in reducing operational costs and emissions while simultaneously enhancing system security and contributing to the development of a sustainable, intelligent future power grid.

## Introduction

In recent decades, the rapid increase in global energy demand has presented significant challenges for modern power systems, necessitating the utilization of all available generation sources, including thermal, hydroelectric, and renewable resources^1,2^. Heavy dependence on fossil fuels has not only depleted these resources but also driven up fuel costs and greenhouse gas emissions, intensifying concerns about sustainability and environmental impacts^1^. Addressing the previous challenges requires energy systems to maximize the efficient use of conventional resources under varying conditions while integrating RES^3^. Economic Dispatch (ED) plays a pivotal role in coordinating generation units to ensure reliable, cost-effective, and environmentally sustainable operations^4^.

The DEED is an optimization problem proposed to minimize the total generation cost over 24 h by optimizing fuel consumption and reducing pollutant emissions. The objective is achieved by optimally adjusting the power output of each generator based on the system load demand, specific fuel cost, and emission characteristics of the generators. Several operational constraints must be satisfied throughout the DEED process, including generation capacity limits, power balance, and ramp rate limits. Additional constraints may include emission limits and prohibited operating zones^1,3,5^.

Various optimization techniques are considered in the literature to address the DEED, including traditional methods such as the gradient-based approach^6^, nature-inspired algorithms like PSO^7^, Teaching-Learning-Based Optimization (TLBO)^8,9^, Grey Wolf Optimization (GWO)^10^, Ant Lion Optimization (ALO)^11^, and Artificial Bee Colony (ABC)^12^. Additionally, hybrid strategies, such as the combination of PSO and Termite Colony Optimization (TCO), are implemented to further enhance performance^13^. Moreover, artificial intelligence-based approaches, including Neural Networks (NN)^6,14^, have also been investigated to solve the DEED problem effectively.

Currently, there is rapid progress in the development and utilization of RES, driven by the global effort to reduce environmental emissions associated with fossil fuel consumption^3^. As RES are clean and sustainable, the adoption of RES also supports the growing demand for a continuous energy supply^3^. Consequently, the integration of RES, particularly solar and wind power, into ED operations has garnered significant attention^3,15–19^. However, RES are inherently intermittent due to the dependence on climatic conditions. For example, solar power generation is directly affected by solar irradiance, which fluctuates throughout the day and across different seasons. In contrast, wind power is not confined to specific periods, as wind speed does not follow a fixed daily cycle but remains influenced by meteorological fluctuations^17,18,20^.

RES, including wind and PV power generation in DEED, are generally considered non-dispatchable due to dependence on environmental factors, resulting in variable and uncontrollable power output. In such cases, RES are typically treated as a priority or must-take unit in DEED models to minimize fuel consumption and reduce emissions. In contrast, conventional thermal units are dispatched to meet the remaining load and maintain system reliability, enabling the effective integration of RES into power systems while adhering to operational constraints and enhancing overall economic efficiency^16,17^. However, integrating advanced forecasting techniques, Energy Storage (ES) systems, and smart grid technologies has facilitated the partial transformation of RES into dispatchable resources. ES enables the integration of wind and PV generation into the grid by storing excess energy generated during peak production periods and releasing energy during times of high demand, thereby enhancing stability and reliability. However, the storing and releasing energy process is complex and involves significant costs^18,19^.

Numerous studies have been conducted to integrate RES with conventional thermal units for optimal ED. For example, several research works have developed multi-objective optimization frameworks aimed at managing the challenges posed by the stochastic variability and uncertainty inherent in RES. For instance, in Ref.^21^, the multi-objective Real-coded Non-dominated Sorting Genetic Algorithm II (R-NSGA-II) has been introduced to optimize power generation from solar, wind, and hydro sources. Results confirm that integrating RES and utilizing the R-NSGA-II approach not only enhances sustainability but also achieves cost-effectiveness in power dispatch, balancing economic and environmental goals. In Ref.^22^, an improved PSO algorithm is proposed for the ED of power systems integrating wind and PV power. The results highlight improved sustainability through optimized resource allocation.

Other studies have highlighted the capability of hybrid and enhanced optimization techniques to strengthen the integration of RES while addressing additional complexities inherent in modern power systems. For example, Ref.^23^ highlights that integrating solar farms into power systems substantially reduces pollutant emissions while enhancing overall system reliability. Moreover, the study introduces a hybrid Differential Evolution (hDE)–Multi-Objective Flower Pollination Algorithm (MOFPA) as an ED approach combining both generation flexibility and the reliability of RES. The Enhanced Cheetah Optimizer Algorithm (ECOA) is introduced in Ref.^15^ for dynamic ED, aiming to reduce energy distribution costs through effective integration of RES and demand-side management. A hybrid DEED model combining RES, including wind, solar, and thermal generation, was developed in Ref.^20^, employing an improved Moth-Flame Optimization technique based on the Position Disturbance Updating strategy (MFO-PDU). The model is designed to minimize fuel expenses and emissions while boosting RES penetration. Ref.^24^ introduces an improved version of the Sailfish Optimization (SFO) algorithm to enhance search efficiency and convergence speed. The approach effectively models the stochastic nature of wind energy using the Weibull distribution within the DEED framework, representing a significant advancement in the DEED field and contributing to the broader objective of sustainable energy management in power systems. Ref.^25^ proposes a novel hybrid optimization framework that combines PSO with a Modified Shuffled Frog Leaping Algorithm (MSFLA) to effectively address the DEED problem, while incorporating demand response programs alongside ES and RES, thereby enhancing system flexibility and operational efficiency. The proposed strategy achieves significantly improved trade-off solutions between fuel cost and emission objectives with superior convergence performance.

More recent studies have been directed toward advanced multi-objective and stochastic optimization frameworks, aiming to maximize the contribution of RES in strengthening the overall performance of ED. For instance, in Ref.^26^, the Multi-Objective Multiverse Optimization (MOMVO) algorithm is introduced to address the dynamic load dispatch problem, considering both cost and emission objectives in the presence of hybrid RES, aiming to reduce overall costs and emissions. Ref.^27^ demonstrates that the use of the Equilibrium Optimizer (EO) significantly improves the optimization process by integrating RES and Plug-in Electric Vehicles (PEVs), resulting in reduced fuel costs and emissions in power systems.

The reported literature^15,20–27^ favors the application of RES in power systems, as RES can minimize the costs and pollution levels. Studies further emphasized the use of diverse optimization approaches to address the increased complexity arising from the incorporation of RES. Moreover, the functionality of RES for the power systems with limited and/or fuel shortage has not been thoroughly investigated.

To address the previous gap, the current research work has evolved to analyze the performance of the power system under the operating scenario of fuel shortage and the inclusion of RES. The previous work of the authors in the area of fuel shortage represents the basis for the present study^1,28^. In Ref.^1^, TLBO was implemented on the IEEE 39-bus system with 10 generating units, focusing on a single-unit fuel shortage scenario. The outcomes validate the proposed DGC approach as both efficient and straightforward for handling ED under fuel limitations. In Ref.^28^, four algorithms, WaOA, Artificial Gorilla Troops Optimization (AGTO), PSO, and Genetic Algorithm (GA), were implemented to evaluate the proposed DEED model on the IEEE 30-bus system under various fuel shortage scenarios. The results confirm the efficiency and simplicity of the proposed DGC approach across different scenarios. Table 1 presents a comprehensive comparison between the existing literature and the proposed research in this study, with particular emphasis on fuel shortage conditions and the integration of RES.

**Table 1:** Summary of the comparison between existing literature and the proposed study for ED, highlighting fuel shortage conditions and RES integration.

Reference	Year	Case study	Utilized algorithm	Generation limits	Fuel shortage condition	RES	Main contribution
Ref.^15^	2024	10-20 units	ECOA	Fixed	✘	**✓**	Reducing cost and emissions via RES, and addressing the unpredictability of RES
Ref.^20^	2021	5-10-15 units	MFO-PDU	Fixed	✘	**✓**	Reducing cost and emissions via RES, and managing complexity in RES integration
Ref.^21^	2023	IEEE 30-bus	MOMVO	Fixed	✘	**✓**	Reducing cost and emissions via RES, and managing complexity in RES integration
Ref.^22^	2023	IEEE 30-bus	Improved PSO	Fixed	✘	**✓**	Reducing cost and emissions via RES, and managing complexity in RES integration
Ref.^23^	2024	10 units	hDE–MOFPA	Fixed	✘	**✓**	Reducing cost and emissions via RES, and mitigating uncertainty and operational risks in RES
Ref.^24^	2021	5-10 units	SFO	Fixed	✘	**✓**	Reducing cost and emissions via RES, and managing complexity in RES integration
Ref.^25^	2025	10 units	PSO-MSFLA	Fixed	✘	**✓**	Reducing cost and emissions via RES, and development of a hybrid optimization algorithm for DEED with RES and ES integration
Ref.^26^	2023	6-10-11 units	MOMVO	Fixed	✘	**✓**	Reducing cost and emissions via RES, and managing complexity in RES integration
Ref.^27^	2022	10-20 units	EO	Fixed	✘	**✓**	Reducing cost and emissions via RES, and managing complexity in RES and PEVs integration
Ref.^1^	2025	IEEE-39 bus	TLBO	Dynamic	**✓**	✘	Optimizing the performance of ED during fuel shortages
Ref.^28^	2025	IEEE 30-bus	WaOA, AGTO, PSO, and GA	Dynamic	**✓**	✘	Optimizing the performance of ED during fuel shortages
Proposed work	-	10 units	MPA, WaOA, and PSO	Dynamic	✓	✓	Optimizing the performance of ED during fuel shortages, managing complexity in RES integration, and using RES to offset power deficit from fuel shortages

The article examines the behavior of DEED under fuel shortage at two generating units, along with the integration of non-dispatchable wind and PV power generation. The DEED process is enhanced by applying DGC. The proposed approach is evaluated using a system comprising 10 units, employing the MPA, WaOA, and PSO algorithms for DEED. The proposed study aims to ensure that load demands are continuously fulfilled while minimizing both operational costs and emissions, even in scenarios of fuel shortages. The article claims to have the following contributions:

- Carrying out a comprehensive comparison between MPA, WaOA, and PSO optimizers for solving DEED problems.
- Introducing a simple and reliable DEED model for a power system, combining wind and PV systems with thermal units operating under limited fuel constraints.
- Enhancing the DEED process by effectively managing limited fuel conditions through DGC, determined based on the available fuel quantities and RES power generation to fulfill the load requirements.

The remainder of this article is structured as follows: ‘‘DEED Mathematical Formulation’’ presents detailed information on the targeted DEED, including the objective function and associated constraints. Sections ‘‘WaOA’’ and ‘‘MPA’’ contain a detailed explanation of the proposed optimization methods. Sections ‘‘System under Concern’’ and ‘‘Methodology’’ present the system, the cases under investigation, and the applied methodology, respectively. The section ‘‘Results’’ provides a detailed discussion of the results. Section ‘‘Power Security against Fuel Cost and Emission’’ provides a concise overview of the contribution to energy security and energy economics. Finally, ‘‘Conclusion’’ concludes with a summary of the key findings and suggestions for future work.

## DEED mathematical formulation

### Objective function

In the present study, PV and wind power plants are considered pre-existing, and their capital costs are not included. The marginal cost of electricity from these RES is assumed to be zero, as no fuel cost is incurred. Moreover, operation and maintenance costs are not taken into account for either RES or thermal power plants, in order to ensure a consistent and fair comparison framework. While it is possible to include annual maintenance or operational costs, such additions have a negligible impact on the optimal dispatch solution due to the relatively low magnitude of these costs compared to the total system operating cost. RES generation is therefore prioritized in the dispatch schedule, and any remaining load demand is supplied by conventional thermal units. No fixed or capacity-dependent costs are applied to RES generation in the objective function.

The DEED aims to minimize the total daily generation cost, $$\:{T}_{C}\:$$(in $/h), comprising fuel and emission costs, as described by the following equationss^9,24,26^:

1 $$\:{T}_{C}=\:{F}_{C}+{E}_{C}$$

2 $$\:{F}_{C}=\:\sum\:_{i=1}^{N}\:\left({x}_{i}{\left({P}_{i}\right)}^{2}+{y}_{i}{P}_{i}+{z}_{i}+\left|{\theta\:}_{i}\:\mathrm{sin}\left({\psi\:}_{i}\left({P}_{i,\:min}-{P}_{i}\right)\right)\right|\right)\:$$

3 $$\:{E}_{C}={h}_{i}*\left({\sum\:}_{i=1}^{N}\:\:\left({\alpha\:}_{i}{\left({P}_{i}\right)}^{2}+{\beta\:}_{i}{P}_{i}+{\gamma\:}_{i}+{\eta\:}_{i}\:exp\left({\varphi\:}_{i}{P}_{i}\right)\right)\right)$$

4 $$\:{h}_{i}=\frac{{F}_{C}\left({P}_{i,max}\right)}{{E}_{C}\left({P}_{i,max}\right)}=\frac{\left(\:{x}_{i}{\left({P}_{i,max}\right)}^{2}+{y}_{i}{P}_{i,max}+{z}_{i}+\left|{\theta\:}_{i}\:{sin}\left({\psi\:}_{i}\left({P}_{i,\:min}-{P}_{i,max}\right)\right)\right|\right)}{\left({\alpha\:}_{i}{\left({P}_{i,max}\right)}^{2}+{\beta\:}_{i}\:{P}_{i,max}+{\gamma\:}_{i}+{\eta\:}_{i}\:exp\left({\varphi\:}_{i}{P}_{i,max}\right)\right)}$$

$\:{F}_{C}\:$represents the total fuel cost, $\:{E}_{C}\:$denotes the emission cost, $\:{P}_{i}$​ signifies the generated power of unit $\:i$ (in MW), and *N* represents the number of units. The variables $\:{x}_{i},\:{y}_{i},\:\mathrm{a}\mathrm{n}\mathrm{d}\:\:{z}_{i}$ are correspond to the coefficients of fuel cost, while $\:{h}_{i}$ represents the penalty factor. $\:{\theta\:}_{i},\:$and $\:{\psi\:}_{i}\:$expresses the coefficient that characterizes the valve-point loading effect. Additionally, $\:{\alpha\:}_{i},\:{\beta\:}_{i},\:{\gamma\:}_{i}$, $\:{\eta\:}_{i}$, and $\:{\varphi\:}_{i}\:$denote the emission coefficients. Furthermore,$\:\:{P}_{i,\:min}$, and $\:{P}_{i,max}\:$indicates the minimum and maximum generation limit for unit $\:i$^9,24,26^.

### Constraints

The objective function is subject to several constraints, including the power balance constraints specified in Eqs. 5 and 6, the generation limits outlined in Eqs. 7 and 8, and the ramp rate limits stated in Eqs. 9^9,24,26^.

### Power balance

To maintain system balance, the total generated power from thermal and RES must match the load demand plus transmission losses. The mathematical expression is given below^9,24,26^:

5 $$\:\sum\:_{i=1}^{N}\:{P}_{i}+\sum\:_{k=1}^{{N}_{W}}\:{P}_{k,W}+\sum\:_{m=1}^{{N}_{PV}}\:{P}_{m,PV}={P}_{L}+{P}_{D}$$

6 $$\:{P}_{L}=\sum\:_{i=1}^{N}\:\sum\:_{j=1}^{N}\:{P}_{i}{B}_{ij}{P}_{j}+\sum\:_{i=1}^{N}\:{B}_{0i}{P}_{i}+{B}_{00}$$

where $\:{P}_{D}$ denotes the load demand (MW), $\:{P}_{L}\:$ represents the power losses (MW),$\:\:{P}_{k,W}$​ and $\:{P}_{m,PV}$ denote the power generated from $\:k$ and *m* wind and PV of units, respectively. $\:{N}_{W}$ and$\:\:{N}_{PV}$ represent the number of wind and PV units. The terms $\:{B}_{ij},{B}_{oi}{,\:\mathrm{a}\mathrm{n}\mathrm{d}\:B}_{oo}$ represent the transmission line loss coefficients^9,24,26^.

The power balance equality constraint is handled using a penalty-based fitness function. Any deviation between total generated power and load demand is penalized proportionally, leading to an increased fitness value. As the problem is formulated as a minimization task, infeasible solutions with power imbalance are naturally discarded, ensuring that the final solution strictly satisfies the power balance constraint.

### Generating power limits

For optimal efficiency and minimal heat rate, generating units must operate within assigned operating limits as given below^9,24,26^.

7 $$\:{P}_{i,\:min}\le\:{P}_{i}\le\:{P}_{i,max},\:\:i=1,\dots\:,N$$

Generation limits are enforced through bounded initialization and feasibility-preserving boundary control. Candidate solutions are generated within unit-specific lower and upper limits, ensuring that all positions remain within permissible generation ranges throughout the optimization process.

### Dynamic generation capacity

To mitigate risk situations such as fuel shortages, the DGC technique is applied. This approach defines realistic hourly generation limits based on the available fuel, thereby preventing infeasible power outputs that may cause system imbalance. As expressed in Eq. 2, the generated power is linked to the available fuel through the generation cost under the assumption of a constant fuel price, which enables the determination of the maximum allowable generation for each hour. Since Eq. 2 represents a nonlinear cost function, analytical techniques such as the quadratic formula are no longer adequate for obtaining a solution^1^. Therefore, a numerical approach was employed using MATLAB’s fsolve function^30^, providing an effective solution for complex nonlinear equations and an accurate determination of the roots when a reasonable initial guess is provided. As a result, the value of $\:{P}_{i}$ can be computed, enabling the determination of the corresponding power output for a given fuel quantity. For fuel shortages, generation capacity is adjusted and treated as a variable dependent on the available fuel, referred to as DGC, as outlined below:

8 $$\:\left\{\begin{array}{c}{P}_{i,max}^{n}={P}_{i,max}^{o},{P}_{i,min}^{n}={P}_{i,min}^{o},\:\:\:\:\:\:\:\:\:\:\:\:\:if\:{P}_{i}\ge\:{P}_{i,max}^{o}\\\:{\:{P}_{i,max}^{n}=\:\:P}_{i},{P}_{i,min}^{n}={P}_{i,min}^{o},\:{\:\:\:if\:\:{P}_{i,min}^{o}\le\:P}_{i}\le\:{P}_{i,max}^{o}\\\:{P}_{i,max}^{n}={P}_{i,min}^{n}=0,\:\:\:\:\:\:\:\:\:\:\:\:\:\:\:\:\:\:\:\:\:\:\:\:\:\:\:\:\:\:\:\:\:\:\:\:{if\:\:P}_{i}\le\:{P}_{i,min}^{o}\end{array}\right.$$

where $\:{P}_{i,min}^{o},\:\:\mathrm{a}\mathrm{n}\mathrm{d}\:{P}_{i,max}^{o}$ denote the previous generation limits while$\:{\:P}_{i,min}^{n},{P}_{i,max}^{n}$​ represent the updated limits^1,28^.

### Ramp rate limit

To maintain the safe operation of each thermal unit, the rate of increase or decrease in the output power must not exceed specified limits. The ramp rate constraint is mathematically represented as follows:

9 $$\:\left\{\begin{array}{c}{P}_{i}\left(t\right)-{P}_{i}(t-1)\le\:{UR}_{i}\\\:{P}_{i}\left(t-1\right)-{P}_{i}\left(t\right)\le\:{DR}_{i}\end{array}\right.$$

where $\:{P}_{i}\left(t\right)$ and $\:{P}_{i}(t-1)$ denote the current and previous output power of generating unit $\:i$, respectively.$\:\:U{R}_{i}$ and $\:D{R}_{i}$ indicate the upper and lower bounds of output power fluctuations, respectively^9,24,26^.

Ramp-rate constraints are handled using a hybrid repair–penalty approach. Newly generated solutions are first adjusted to satisfy up- and down-ramp limits relative to the previous operating point, while any remaining violations are penalized during fitness evaluation, ensuring feasibility and stable convergence.

### WaOA

WaOA models the feeding behavior, migration, and predator evasion strategies of walruses^31^. WaOA’s search mechanism is especially effective in high-dimensional environments, enabling the avoidance of local optima and resulting in superior solutions compared to alternative approaches^31^. The position update process in WaOA is structured into three primary phases as follows:

Exploration phase: Involves the movement of walruses toward the fittest individual, facilitating the search for superior feeding areas. Position updates are mathematically expressed as follows:

10 $$\:{X}_{i}^{p1}={X}_{i}+{rand}_{1}\left({X}_{best}-{I}_{1}{X}_{i}\right)$$

The updated position is represented by:

11 $$\:{X}_{i}=\left\{\begin{array}{c}{X}_{i}^{p1},{\:\:\:\:\:\:\:\:f}_{i}^{p1}<{f}_{i}\\\:{X}_{i\:\:},\:\:\:\:\:\:\:\:\:\:\:\:\:\:\:\:else\end{array}\right.$$

$\:{X}_{i}^{p1}$ refers to the new position of the *i*-th walrus, and $\:{X}_{i}$​ indicates the prior position. $\:{rand}_{1}$​ is a random number generated within [0, 1], and $\:{X}_{best}$​ signifies the most optimal solution identified. $\:{I}_{1}$​ is a value randomly drawn from the range [1, 2], while $\:{f}_{i}^{p1}$ and $\:{f}_{i}$ ​denote the fitness at the new and old positions, respectively^32,33^.

Migration phase: Involves the movement of walruses toward a randomly selected walrus. The behavior of walruses during the current stage can be mathematically expressed as follows:

12 $$\:{X}_{i}^{p2}=\left\{\begin{array}{c}{X}_{i}+{rand}_{2}\left({X}_{rd}-{I}_{1}{X}_{i}\right),\:\:{f}_{rd}<{f}_{i}\\\:{X}_{i}+{rand}_{2}\left({X}_{i}-{X}_{rd}\right),\:\:\:\:\:\:\:\:\:\:\:\:\:\:\:\:\:\:else\end{array}\right.$$

The updated position is represented by:

13 $$\:{X}_{i}=\left\{\begin{array}{l}{X}_{i}^{p2},\:\:{f}_{i}^{p2}<{f}_{i}\\\:{X}_{i\:\:\:},\:\:\:\:\:\:\:\:\:\:else\end{array}\right.$$

$\:{X}_{i}^{p2}$ ​indicates the new position of the *i*-th walrus, while $\:{X}_{rd}$ ​refers to the position of a randomly selected walrus. The fitness at $\:{X}_{rd}$ position is denoted by $\:{f}_{rd}$​. $\:{rand}_{2}\:$is a random number generated within the interval [0, 1]. $\:{f}_{i}^{p2}$​ represents the fitness at the new position $\:{X}_{i}^{p2\:}$^32,33^.

Exploitation phase: Involves the evasion and defense behavior of walruses against predators. The mathematical representation of the behavior is given below:

14 $$\:{X}_{i}^{p3}={X}_{i}+\left(l{b}_{\left(t\right)}+\left(u{b}_{\left(t\right)}-{rand}_{3}\:l{b}_{\left(t\right)}\right)\right)$$

15 $$\:\left\{\begin{array}{l}l{b}_{\left(t\right)}=\frac{lb}{t}\\\:u{b}_{\left(t\right)}=\frac{ub}{t}\end{array}\right.$$

The new position after one iteration is represented by:

16 $$\:{X}_{i,new}=\left\{\begin{array}{l}{X}_{i}^{p3},{\:\:\:f}_{i}^{p3}<{f}_{i}\\\:{X}_{i},\:\:\:\:\:\:\:else\end{array}\right.$$

**Fig. 1:**
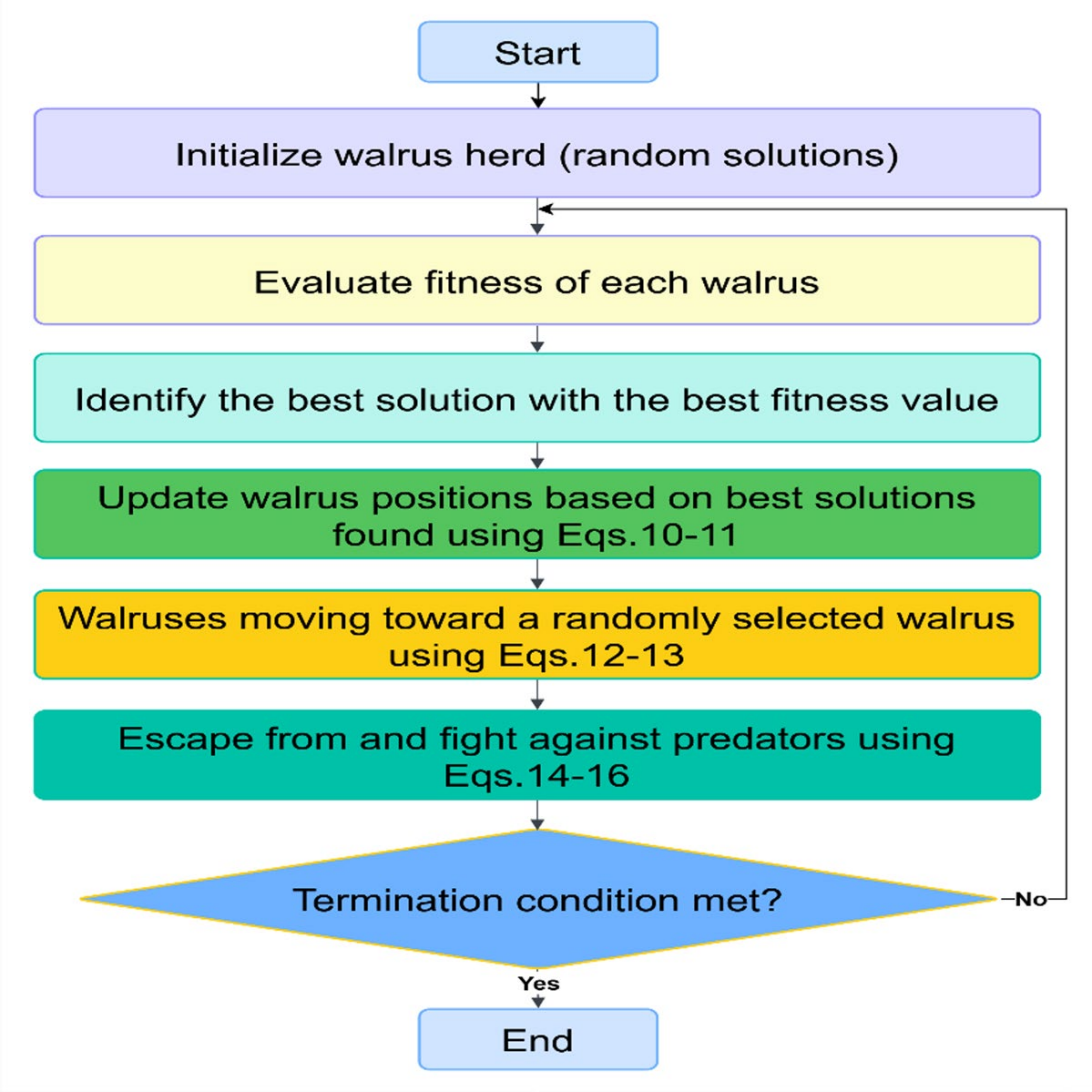
Operational flowchart of the WaOA technique^30^.

In the third phase, $\:{X}_{i}^{p3}$​ signifies the updated location of the *i*-th walrus. $\:{rand}_{3}$​ is a random number generated within [0, 1], and $\:{X}_{i,new}$ denotes the final position of the walrus. The terms $\:lb$ and $\:ub$ correspond to the lower and upper position limits. $\:{f}_{i}$ ​and $\:{f}_{i}^{p3}$ ​represent the fitness values at $\:{X}_{i}$ ​ and $\:{X}_{i}^{p3}$​, respectively^32,33^. Figure 1 illustrates the flowchart detailing the main steps of the WaOA^30^.

### MPA

MPA is a nature-inspired optimization method based on the foraging strategies of marine predators, primarily utilizing Levy and Brownian movement patterns and demonstrating successful application in diverse research domains^34^. The MPA is structured into three primary phases as follows^35–37^:

Exploration phase: Occurring in the early third of iterations under high velocity ratios, the phase involves prey movement guided by Brownian motion, leading to the following updates:

17 $$\:\overrightarrow{{step\_size}_{i}\:\:}=\overrightarrow{{R}_{B}\:\:}\:\:\otimes\:\:\left(\overrightarrow{{E}_{i}\:\:}-\overrightarrow{{R}_{B}\:\:}\:\otimes\:\:\:\overrightarrow{{Prey}_{i}\:\:}\right)$$

18 $$\:\overrightarrow{{Prey}_{i}\:\:}=\overrightarrow{{Prey}_{i}\:\:}+P\times\:\overrightarrow{R\:\:}\otimes\:\overrightarrow{{step\_size}_{i}\:\:}$$

where $\:P$ =0.5, $\:\overrightarrow{R\:\:}$is a vector of uniformly distributed random numbers within the range [0, 1], and $\:\overrightarrow{{R}_{B}\:}\:$is a vector of random values generated according to a normal distribution representing Brownian motion.$\:\:\overrightarrow{{E}_{i}\:\:}$represents the Elite matrix^35,36^.

Intermediate phase: Taking place in the middle third of the iterations at equal velocity, the population is split evenly—50% for exploration and 50% for exploitation^35–37^. The first 50% of the population is updated according to the following procedure:

19 $$\:\overrightarrow{{step\_size}_{i}\:\:}=\overrightarrow{{R}_{L}\:\:}\:\:\otimes\:\:\left(\overrightarrow{{E}_{i}\:\:}-\overrightarrow{{R}_{L}\:\:}\:\otimes\:\:\:\overrightarrow{{Prey}_{i}\:\:}\right)$$

20 $$\:\overrightarrow{{Prey}_{i}\:\:}=\overrightarrow{{Prey}_{i}\:\:}+P\times\:\overrightarrow{R\:\:}\otimes\:\overrightarrow{{step\_size}_{i}\:\:}$$

The second 50% of the population is updated according to the following procedure:

21 $$\:\overrightarrow{{step\_size}_{i}\:\:}=\overrightarrow{{R}_{B}\:\:}\:\:\otimes\:\:\left(\overrightarrow{{R}_{B}\:\:}\otimes\:\overrightarrow{{E}_{i}\:\:}-\:\:\overrightarrow{{Prey}_{i}\:\:}\right)$$

22 $$\:\overrightarrow{{Prey}_{i}\:\:}=\overrightarrow{{E}_{i}\:\:}+P\times\:CF\otimes\:\overrightarrow{{step\_size}_{i}\:\:}$$

23 $$\:CF={\left(1-\frac{itr}{Maxitr}\right)}^{\left(\frac{2\times\:itr}{Maxitr}\right)}$$

where $\:\overrightarrow{{R}_{L}\:}$​ is determined using the principles of Levy flight. The $\:CF$ parameter dynamically controls the step length in the MPA. $\:itr$ specifies the current iteration, while $\:Maxitr$ defines the total number of iterations^35–37^.

Exploitation phase: Occurring in the last third of iterations under low velocity ratios, the phase involves prey movement guided by Levy motion, leading to the following updates:

24 $$\:\overrightarrow{{step\_size}_{i}\:\:}=\overrightarrow{{R}_{L}\:\:}\:\:\otimes\:\:\left(\overrightarrow{{R}_{L}\:\:}\otimes\:\overrightarrow{{E}_{i}\:\:}-\:\:\overrightarrow{{Prey}_{i}\:\:}\right)$$

25 $$\:\overrightarrow{{Prey}_{i}\:\:}=\overrightarrow{{E}_{i}\:\:}+P\times\:CF\otimes\:\overrightarrow{{step\_size}_{i}\:\:}$$

Marine predators are naturally influenced by Fish Aggregating Devices (FADs), spending most of the time near FADs. During the remaining time, longer movements occur across various dimensions to explore areas with a wider distribution of prey^35–37^. The effect of FADs can be formulated mathematically as follows:

26 $$\:\overrightarrow{{Prey}_{i}\:\:}=\left\{\begin{array}{c}\overrightarrow{{Prey}_{i}\:\:}+CF\:\left[\overrightarrow{{X}_{mn}\:}+\overrightarrow{R\:\:}\otimes\:\left(\overrightarrow{{X}_{mx}\:}-\overrightarrow{{X}_{mn}\:}\right)\right]\:\otimes\:\:\:\overrightarrow{U\:},\:\:\:\:\:\:\:\:\:if\:\:\:r\le\:FADs\:\\\:\overrightarrow{{Prey}_{i}\:\:}+\:\left[FADs\:\left(1-r\right)+r\right]\:\left(\overrightarrow{{Prey}_{r1}\:}-\overrightarrow{{Prey}_{r2}\:}\right),\:\:\:\:\:\:\:\:\:if\:\:\:r>FADs\end{array}\right.$$

**Fig. 2:**
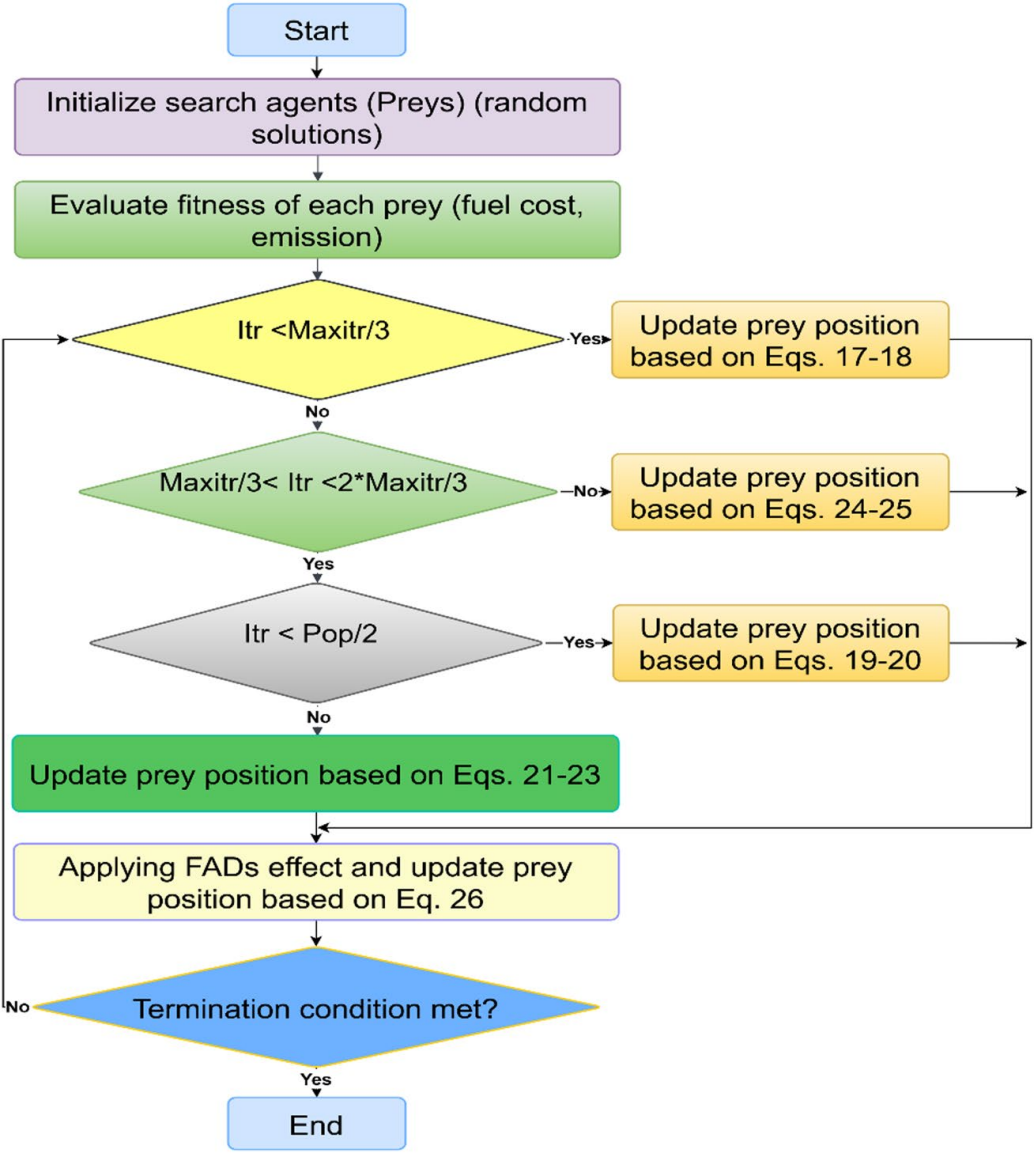
MPA flow chart based on ED^32^.

$\:FADs\:$= 0.2 reflects the influence level of FADs on the optimization. $\:\overrightarrow{U\:}$represents a binary vector with values of 0 and 1. The term$\:\:r$ is a random number uniformly distributed in [0, 1], while $\:{X}_{mx}\:$and $\:{X}_{mn}$ indicate the maximum and minimum limits of the problem dimensions. $\:{Prey}_{r1}$ and $\:{Prey}_{r2}\:$refer to randomly selected entries in the prey matrix^35–37^. Figure 2 illustrates the flowchart outlining the key steps involved in implementing the MPA method for ED^32^.

### System under Concern

**Fig. 3:**
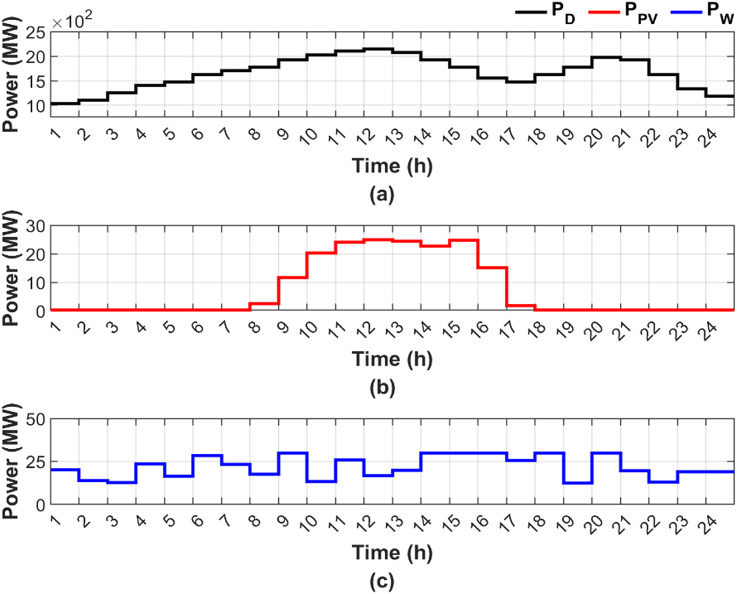
Load profile and renewable energy output characteristics for the considered system^38,39^.

The analysis is performed on a 10 thermal units system with a total installed capacity of 2368 MW. Additionally, PV and wind stations are integrated into the proposed system. The 10-unit system achieves a practical balance between modeling complexity and computational tractability, thereby allowing equitable comparison to present the research gap. The application of the 10-unit system facilitates the evaluation of the proposed method’s capability to manage larger and more complex systems under fuel shortage conditions. Moreover, the integration of RES enhances the realism of the case study and reflects the operational challenges of modern power systems. The specifications of the 10 units are detailed in Table 2, while the loss coefficients are presented in Table 3^38,39^. Figure 3 presents the forecasted power output of RES and the corresponding load demand^37,38^. Although the temporal variations of PV power, wind power, and hourly load demand are already illustrated graphically, presenting the same data in tabular form is essential to ensure numerical transparency, facilitate reproducibility, and enable precise quantitative analysis. Accordingly, Table 4 presents the numerical values of the available PV power, wind power, and hourly load demand over the scheduling horizon.

**Table 2:** Generating unit test data for the 10-unit system^35,36^.

Gen. unit	$$\:{x}_{i}$$ ($/MW^2^.h)	$$\:{y}_{i}$$ ($/MW.h)	$$\:{z}_{i}$$ ($/h)	$$\:{\theta\:}_{i}$$ ($/h)	$$\:{\psi\:}_{i}$$ red/MW	$$\:{\alpha\:}_{i}$$ (lb/MW^2^.h)	$$\:{\beta\:}_{i}$$ (lb/MW.h)	$$\:{\gamma\:}_{i}$$ (lb/h)	$$\:{\eta\:}_{i}$$ (lb/h)	$$\:{\varphi\:}_{i}$$ 1/MW	$$\:{UR}_{i}$$ (MW/h)	$$\:{DR}_{i}$$ (MW/h)	*P*_i, min_ (MW)	*P*_i, max_ (MW)
1	0.1524	38.5397	786.7988	450	0.041	0.0312	2.4444	103.3908	0.5035	0.0207	80	80	150	470
2	0.1058	46.1591	451.3251	600	0.036	0.0312	2.4444	103.3908	0.5035	0.0207	80	80	135	470
3	0.0280	40.3965	1049.998	320	0.028	0.0509	4.0695	300.3910	0.4968	0.0202	80	80	73	340
4	0.0354	38.3055	1243.531	260	0.052	0.0509	4.0695	300.3910	0.4968	0.0202	50	50	60	300
5	0.0211	36.3278	1658.570	280	0.063	0.0344	3.8132	320.0006	0.4972	0.0200	50	50	73	243
6	0.0179	38.2704	1356.659	310	0.048	0.0344	38.132	320.0006	0.4972	0.0200	50	50	57	160
7	0.0121	36.5104	1450.705	300	0.086	0.0465	3.9023	330.0056	0.5163	0.0214	30	30	20	130
8	0.0121	36.5104	1450.705	340	0.082	0.0465	3.9023	330.0056	0.5163	0.0214	30	30	47	120
9	0.1090	39.5804	1455.606	270	0.098	0.0465	3.9524	350.0056	0.5475	0.0234	30	30	20	80
10	0.1295	40.5407	1469.403	380	0.094	0.0470	3.9864	360.0012	0.8475	0.0234	30	30	10	55

**Table 3:** Parameters of transmission loss for the 10-unit system^35,36^.

B-loss coefficient									
4.9E − 05	1.4E − 05	1.5E − 05	1.5E − 05	1.6E − 05	1.7E − 05	1.7E − 05	1.8E − 05	1.9E − 05	2.0E − 05
1.4E − 05	4.5E − 05	1.6E − 05	1.6E − 05	1.7E − 05	1.5E − 05	1.5E − 05	1.6E − 05	1.8E − 05	1.8E − 05
1.5E − 05	1.6E − 05	3.9E − 05	1.0E − 05	1.2E − 05	1.2E − 05	1.4E − 05	1.4E − 05	1.6E − 05	1.6E − 05
1.5E − 05	1.6E − 05	1.0E − 05	4.0E − 05	1.4E − 05	1.0E − 05	1.1E − 05	1.2E − 05	1.4E − 05	1.5E − 05
1.6E − 05	1.7E − 05	1.2E − 05	1.4E − 05	3.5E − 05	1.1E − 05	1.3E − 05	1.3E − 05	1.5E − 05	1.6E − 05
1.7E − 05	1.5E − 05	1.2E − 05	1.0E − 05	1.1E − 05	3.6E − 05	1.2E − 05	1.2E − 05	1.4E − 05	1.5E − 05
1.7E − 05	1.5E − 05	1.4E − 05	1.1E − 05	1.3E − 05	1.2E − 05	3.8E − 05	1.6E − 05	1.6E − 05	1.8E − 05
1.8E − 05	1.6E − 05	1.4E − 05	1.2E − 05	1.3E − 05	1.2E − 05	1.6E − 05	4.0E − 05	1.5E − 05	1.6E − 05
1.9E − 05	1.8E − 05	1.6E − 05	1.4E − 05	1.5E − 05	1.4E − 05	1.6E − 05	1.5E − 05	4.2E − 05	1.9E − 05
2.0E − 05	1.8E − 05	1.6E − 05	1.5E − 05	1.6E − 05	1.5E − 05	1.8E − 05	1.6E − 05	1.9E − 05	4.4E − 05

**Table 4:** Hourly values of available PV power, wind power, and load demand used in the considered study^38,39^.

Hour	$\:{P}_{W}\:$(MW)	$\:{P}_{PV}$ (MW)	$\:{P}_{D}\:$ (MW)	Hour	$\:{P}_{W}\:$(MW)	$\:{P}_{PV}$ (MW)	$\:{P}_{D}\:$ (MW)
1	20.25	0	1036	13	19.8	24.381	2072
2	13.95	0	1110	14	30	22.624	1924
3	12.75	0	1258	15	30	24.663	1776
4	23.7	0	1406	16	30	15.107	1554
5	16.5	0	1480	17	25.5	1.7755	1480
6	28.56	0	1628	18	30	0	1628
7	23.25	0	1702	19	12.3	0	1776
8	17.7	2.4452	1776	20	30	0	1972
9	30	11.663	1924	21	19.5	0	1924
10	13.2	20.334	2022	22	12.9	0	1628
11	25.95	24.028	2106	23	18.9	0	1332
12	16.8	24.884	2150	24	18.9	0	1184

## Methodology

The proposed DEED problem is optimized over the entire 24-hour horizon in a single simultaneous run, where all hourly generator outputs are determined jointly. Generation limits, ramp-rate constraints, and power balance are enforced across the full day, with ramp-rate limits inherently applied between consecutive hours. Infeasible solutions violating generation or ramping constraints are excluded, ensuring that the final dispatch schedule is physically feasible and temporally consistent.

The research methodology consists of two phases:

- Phase 1: Comprehensive comparison between MPA, WaOA, and PSO.
- Phase 2: Validating the proposed DEED with a successful candidate optimizer for different operating scenarios.

The objective of phase 1 is to identify the most promising meta-heuristic optimizer from MPA and WaOA. The performance of the candidate optimizers is compared against each other and PSO. PSO is selected for the merits of fast convergence speed, elevated efficiency, and wide applicability for ED.

The control parameters for the MPA, WaOA, and PSO were carefully selected through the Grid Search tuning method. The Grid Search tuning procedure begins by defining discrete candidate values for each control parameter within predefined ranges, after which all possible parameter combinations are systematically evaluated over the resulting search grid^40^. The selection of the optimal parameter values was based on minimizing the average total operating cost obtained over multiple independent runs of the algorithm.

To identify the most promising and robust optimizer from MPA and WaOA, the performance of the proposed optimizers and PSO is evaluated for the system under concern for a 1000 MW load. The comparison between the optimizers MPA, WaOA, and PSO includes the efficiency and effectiveness in fulfilling the load demand level of 1000 MW, with the minimal Total Cost (TC). Moreover, the comparison also involves statistical tools such as Standard Deviation (SD)^1,28^, Confidence Interval (CI) analysis^1,28^, CI Width, and the average execution time across 30 independent runs of the MPA, WaOA, and PSO optimizer^1,28^, providing a sufficient sample size for stable estimation of mean performance and statistical analysis.

The SD quantifies the dispersion of a dataset relative to its mean, indicating how much individual values deviate from the average. A lower SD reflects data consistency, whereas a higher SD indicates greater variability^41,42^. Mathematically, the SD is defined as:

27 $$\:SD=\sqrt{\frac{1}{n-1}{\sum\:}_{d=1}^{n}{\left(\stackrel{-}{x}-{x}_{d}\right)}^{2}}$$

28 $$\:\stackrel{-}{x}=\frac{1}{n}{\sum\:}_{d=1}^{n}{x}_{d}$$

Where $\:{x}_{d}\:$ represents the individual observed data points within the dataset, $\:\stackrel{-}{x}$ denotes the mean of all observations, and $\:n$ indicates the total number of observations in the sample^41,42^.

The CI defines the range within which the true population mean is expected to lie with a specified level of confidence (e.g., 95%). It expresses the precision of the sample mean as an estimate of the population mean^41,42^. Mathematically, the CI is defined as:

29 $$\:CI=\stackrel{-}{x}\pm\:Z*\left(\frac{SD}{\sqrt{n}}\right)$$

Z is the critical value obtained from the standard normal distribution corresponding to the desired confidence level (e.g., 2.045 for a 95% CI and 30 sample)^41,42^.

The final selection of the superior optimizer was based on a comprehensive assessment combining cost minimization, statistical stability, and computational efficiency, rather than relying solely on the best objective function value. A small SD combined with a narrow CI indicates that the algorithm converges consistently toward similar solution qualities, thereby demonstrating the robustness of the optimization algorithm.

In phase 2, four different operational scenarios are considered to test the proposed DEED. They are:

- Scenario 1: DEED without fuel shortage optimized via MPA (Full fuel).
- Scenario 2: DEED with DGC for fuel shortages in units 4 and 9 using MPA (DGC).
- Scenario 3: DEED with integration of RES and fuel shortages in units 4 and 9 via MPA (RES without DGC).
- Scenario 4: DEED with DGC for fuel shortages in units 4 and 9, incorporating RES using MPA (RES with DGC).

In Phase 2, four distinct operational scenarios were designed to rigorously evaluate the proposed DEED method under varying system conditions, with a focus on optimal utilization and management of RES under conditions of fuel shortages through DGC technique. Scenario 1 represents a normal case without fuel shortages, serving as a baseline for comparison. Scenario 2 introduces fuel shortages in units 4 and 9 using the DGC strategy, testing the capability of DGC technique to address critical conditions arising from fuel shortages and to maximize system security in the absence of RES. Scenario 3 considers the integration of RES alongside fuel shortages, assessing the capability of traditional DEED to leverage RES to compensate for power deficits caused by stochastic fuel shortages throughout the day. Scenario 4 combines both DGC for fuel shortages and RES integration, representing the most complex operational condition. This scenario evaluates the DEED with DGC method’s capability to optimally utilize and manage RES in coordination with other units with sufficient fuel, in order to compensate for power deficits and ensure supply security throughout the day. Collectively, the four scenarios are designed to increase operational complexity, from a baseline scenario without fuel shortages to scenarios incorporating fuel shortages conditions and RES integration, thereby providing a comprehensive framework to evaluate the DEED with DGC technique under varying conditions and to demonstrate its robustness, and capability to optimally manage all fuel-shortage scenarios, both with and without RES.

The selection of units was based on capacity considerations, as unit 4 represents one of the highest-capacity generators, whereas unit 9 is among the lowest-capacity units. The contrast was adopted to enable a fair assessment of the proposed method and to demonstrate the capability in managing stations with diverse generation capacities.

## Results

### MPA versus WaOA and PSO

The simulation was conducted using MATLAB on a laptop powered by an Intel Core i7 processor and 16 GB of RAM. A population/particle size of 50 and a maximum of 1000 iterations were employed for all proposed optimizers to ensure consistency in evaluation. For WaOA, the parameter $\:{I}_{1}$ is selected as 1 from the candidate range of [1, 2]. For PSO, the cognitive and social acceleration coefficients (*C*1 and *C*2) were explored within the range [1.5–2.5] with a step size of 0.1, and ultimately set to 1.5. The inertia weight factor was varied within the range [0.4–0.9] with a step size of 0.1 and set to 0.7, while the remaining control parameters were kept constant as previously described.

Table 5 presents the total cost and the power allocation of each unit to meet a total load demand of 1000 MW. Table 6 presents the performance metrics, including SD, CI analysis, and the average execution time across 30 independent runs of the WaOA, MPA, and PSO optimizers.

**Table 5:** Power allocation of each unit to meet a total load demand of 1000 MW.

Method	*P*_1_ (MW)	*P*_2_ (MW)	*P*_3_ (MW)	*P*_4_ (MW)	*P*_5_ (MW)	*P*_6_ (MW)	*P*_7_ (MW)	*P*_8_ (MW)	*P*_9_ (MW)	*P*_10_ (MW)	*P*_L_ (MW)	TC ($)
WaOA	150	135	125.99	120.4	122.87	103.23	80	85.31	52.05	43.42	18.27	90833.95
MPA	150	135	125.44	120.42	122.87	103.75	80	85.31	52.06	43.42	18.27	90833.74
PSO	150	135	139.43	120.42	122.87	105.8	63.99	85.31	52.06	43.42	18.30	90941.43

**Table 6:** Statistical results for the WaOA, MPA, and PSO methods over 30 runs at 1000 MW.

Method	SD	CI	CI Width	Time (s)
WaOA	2.21E+00	[90833.27, 90834.92]	1.65	6.31
MPA	4.38E-01	[90833.72, 90833.76]	0.04	3.03
PSO	2.43E+02	[90853.25, 91034.59]	181.34	2.46

Table 5 shows that WaOA and MPA produce better results compared with PSO, reducing the total cost by almost 0.12%. The close similarity in generation costs between MPA and WaOA reflects the approximate similarity in their nature-inspired search strategies, which balance exploration and exploitation. Compared to PSO, their diverse movement patterns and dynamic exploration-exploitation balance enable them to better navigate complex solution spaces, avoid local optima, and achieve higher-quality solutions.

However, as Table 6 shows, MPA exhibits the highest stability and robustness, evidenced by the CI width. The confidence interval of MPA is lower, at 97.58% and 99.98%, compared to WaOA and PSO, respectively. Moreover, the MPA method achieved reductions of around 80.18% and 99.82% in standard deviation compared to WaOA and PSO, reflecting the consistent performance. MPA achieves superior solution stability through its precisely controlled population movements and finely tuned exploratory steps, enabling accurate convergence toward optimal regions. However, the MPA requires longer computation time compared to PSO, primarily due to the greater complexity of the search operations. The execution time of MPA is nearly 18.8% longer than that of PSO. Meanwhile, MPA has nearly a 52% reduction in computing time compared to WaOA. PSO generally requires less time than both MPA and WaOA because its particle updates are straightforward, relying only on individual and global best positions without additional complex interactions. WaOA, on the other hand, requires the most computational effort because its update mechanisms involve managing multiple individuals in a competitive and cooperative framework, often necessitating additional adjustments to maintain collective dynamics, which increases execution time despite achieving comparable solution costs. According to the comparisons shown in Tables 5 and 6, MPA generally performs better than WaOA and PSO.

Table 6 shows that PSO is relatively faster than MPA; however, the problems of instability and inconsistency remain significant issues, as shown in the SD and CI. The convergence speed of MPA could be enhanced by reducing the population size or the number of iterations.

The reported slight percentage improvement in cost (0.12%) corresponds to a load of 1000 MW, representing less than half of the system’s hourly capacity. Such a saving becomes significant when scaled to larger system loads or aggregated over an entire day, ultimately resulting in substantial monetary benefits across extended operating periods. In the context of fuel shortages and rising fuel prices, even marginal reductions acquire considerable importance. Moreover, the marked decrease in the standard deviation and confidence interval indicates enhanced stability and predictability of the MPA outcomes, providing system operators with reduced risk exposure and more reliable scheduling.

The comparison between MPA, WaOA, and PSO is carried out at several load levels, and the results are almost consistent with the findings presented in Tables 5 and 6. Therefore, MPA is proven to have the best overall performance relative to WaOA and PSO.

### The proposed DEED under numerous operating scenarios

A comprehensive comparison of the generation cost and statistical tools revealed that MPA is the most promising optimizer for implementing DEED in the system under concern. MPA produces the lowest TC, SD, and CI width relative to WaOA and PSO. Therefore, MPA is adopted for validating the proposed DEED with DGC and RES for the power system under study.

The results for each scenario begins with a detailed graphical presentation of the optimal generated power for the two target units, providing a basis for comparison. This is followed by a consolidated view of total generation from all units compared to the hourly load demand, allowing readers to clearly understand both individual plant performance and overall system behavior.

#### Scenario 1: DEED without fuel shortage optimized via MPA

**Fig. 4:**
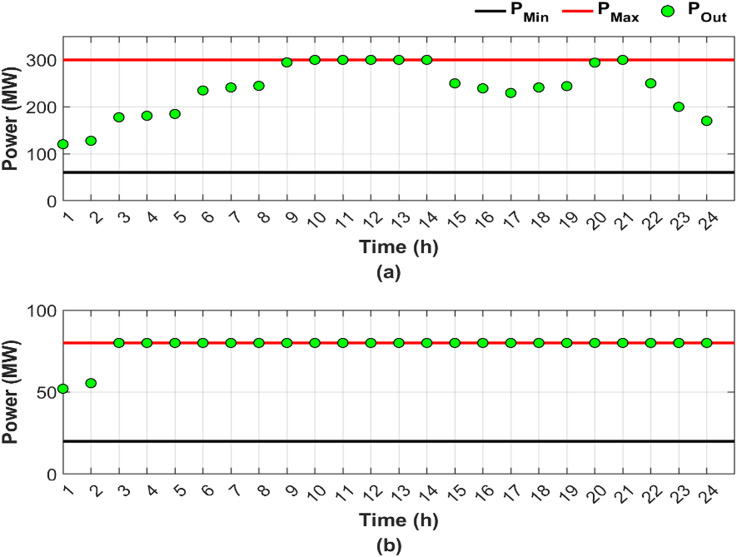
Hourly optimal power generation for Scenario 1: (**a**) unit 4 output and (**b**) unit 9 output (red line: maximum power generation limit, black line: minimum power generation limit).

This scenario represents a normal operation case without fuel shortages and RES, serving as a baseline for comparison, where the objective is to minimize generation costs and emissions by determining the optimal power output, while considering the valve-point loading effect and ramp rate constraints. Figure 4 illustrates the optimal generation power for units 4 and 9 throughout the day, presented in two subfigures: Fig. 4a, b, respectively.

**Fig. 5:**
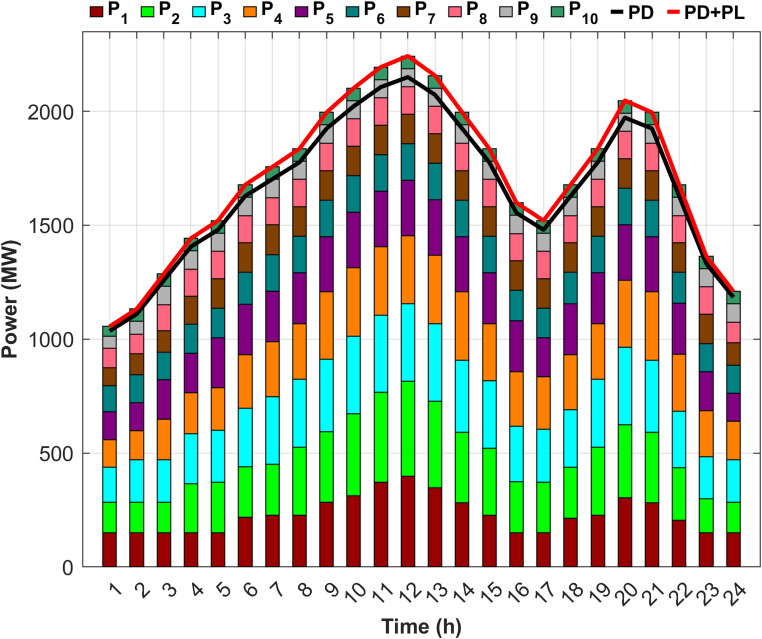
Detailed power generation and demand per hour for Scenario 1.

In Fig. 4, both Fig. 4a, b show that the generation limits remain constant due to the abundance of fuel. A sufficient amount of fuel allows the optimal generated power to change freely based on the required load, leading to a reduction in overall cost. Unit 4 contributed 13.91% of the total required load, primarily due to the high generating capacity, while unit 9 contributed only 4.54% as a result of limited generating capacity. Figure 4a shows that the output of unit 4 varies according to the loading levels, while unit 9 consistently operates at maximum capacity, representing the optimal output level utilized by the ED to minimize both cost and emissions. Figure 5 provides a graphical representation of the optimal generated power for all units.

Figure 5 illustrates the precise balance between generated power, required load, and system losses after determining the optimal generated power from each unit to minimize costs. Figure 5 also illustrates that the first five units account for up to 70% of the total load, attributed to the high generation capacities, whereas the remaining five units, with lower capacities, contribute the remaining 30%.

The detailed numerical results are essential for ensuring transparency and reproducibility, as they allow readers to validate the outcomes of the proposed approach. However, given the size of the dataset to avoid overloading the main text with extensive tabular information, the complete table has been moved to the Appendix. A concise summary is presented here to highlight the most significant findings. Table 10 in Appendix A presents the detailed optimal dispatch results obtained using the MPA, including the generation power and transmission losses for each time period. Table 10 confirms that all generating units operate within the specified generation limits and comply with the power balance constraints. Furthermore, the results show compliance with the ramp rate limits, with the highest observed variations being 80 MW, 50 MW, 30 MW, and 30 MW for units 2, 4, 7, and 8, respectively. The power variation values correspond exactly to their imposed ramp rate limits, thereby confirming that all units remain fully compliant with the operational constraints.

#### Scenario 2: DEED with DGC for fuel shortages in Units 4 and 9 using MPA

This scenario evaluates the efficiency of DEED with DGC while accounting for transmission line losses, the valve-point loading effect, and ramp rate constraints without including RES. The current scenario aims to find the optimal power output for effectively managing fuel quantity limitations while reducing total cost and fulfilling load demand.

Scenario 2 addresses integrated fuel shortage conditions by applying the DGC technique, which adjusts the generation bounds according to the available fuel quantities. To provide additional clarity and facilitate precise interpretation, Table 7 presents the hourly updated generation limits for the targeted units under this scenario.

**Table 7:** Hourly updated generation limits for units 4 and 9 under Scenario 2.

Hour	*P*_4, min_ (MW)	*P*_4, max_ (MW)	*P*_9, min_ (MW)	*P*_9, max_ (MW)	Hour	*P*_4, min_ (MW)	*P*_4, max_ (MW)	*P*_9, min_ (MW)	*P*_9, max_ (MW)
1	60	75.02966	20	22.97344	13	60	300	20	70.61138
2	60	88.7919	20	27.04249	14	60	288.315	20	44.93366
3	60	179.0032	20	31.80464	15	60	265.658	20	37.80269
4	60	228.7907	20	35.08282	16	60	234.4809	20	57.99293
5	60	251.9867	20	50.31222	17	60	192.6935	20	59.11266
6	60	278.4579	20	55.08966	18	60	152.7448	20	35.59163
7	60	287.4176	20	60.94256	19	60	212.8292	20	75.57014
8	60	300	20	73.9797	20	60	244.8522	20	65.03985
9	60	300	20	80	21	60	229.5044	20	35.30268
10	60	298.5149	20	53.28526	22	60	193.6403	20	26.91741
11	60	263.6497	20	41.91491	23	60	152.3643	0	0
12	60	300	20	26.27064	24	0	0	0	0

Table 7 highlights the hourly variations in generation limits caused by fuel shortages. Table 7 also shows that the upper and lower limits are not constant, representing the feature of DGC. DGC is proposed to increase the accuracy in selecting the optimal power while the system is subjected to fuel shortages. In the event of fuel shortages in some units, fixed limits could lead to non-real power, reducing supply security and continuity. Flexible limits ensure that optimization occurs within the context of a realistic power search.

**Fig. 6:**
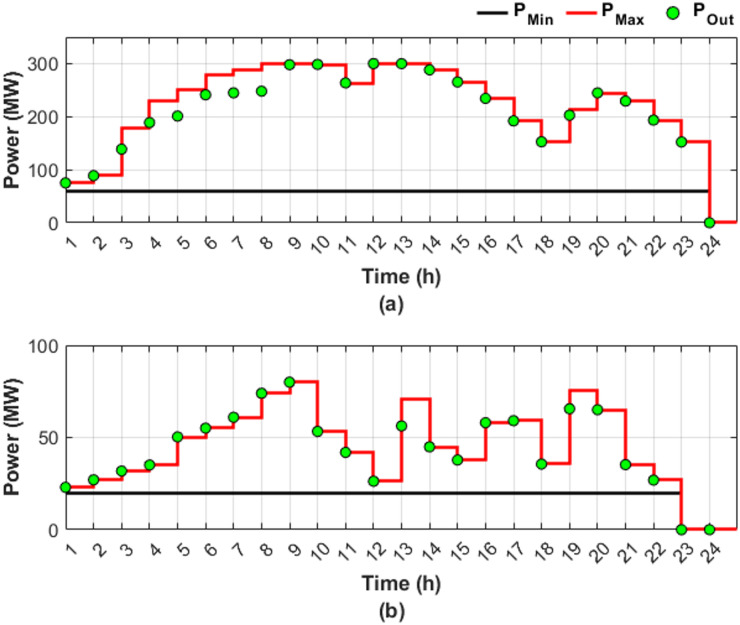
Hourly optimal power generation for Scenario 2: (**a**) unit 4 output and (**b**) unit 9 output (red line: maximum power generation limit, black line: minimum power generation limit).

Figure 6 shows the optimal dispatch output for units 4 and 9 under conditions of fuel shortage, shown in subfigures Fig. 6a, b, respectively.

Figure 6 reveals that unit 4 has greater load sharing than unit 9, similar to Fig. 4 in Scenario 1. Comparing Figs. 4 and 6 reveals that the DGC for the fuel shortage condition resulted in varying the upper and lower limits of both units and hence the produced powers. The limits and output power flexibility are shown clearly in Fig. 6b.

Figure 6 shows that unit 9 has been out of service for the last two hours, followed by unit 4 an hour later, due to complete fuel depletion. The share of the total required load decreased from 13.91% in Scenario 1 to 12.25% in Scenario 2 for unit 4. For unit 9, the load sharing declined from 4.54% in Scenario 1 to 2.53% in Scenario 2.

**Fig. 7:**
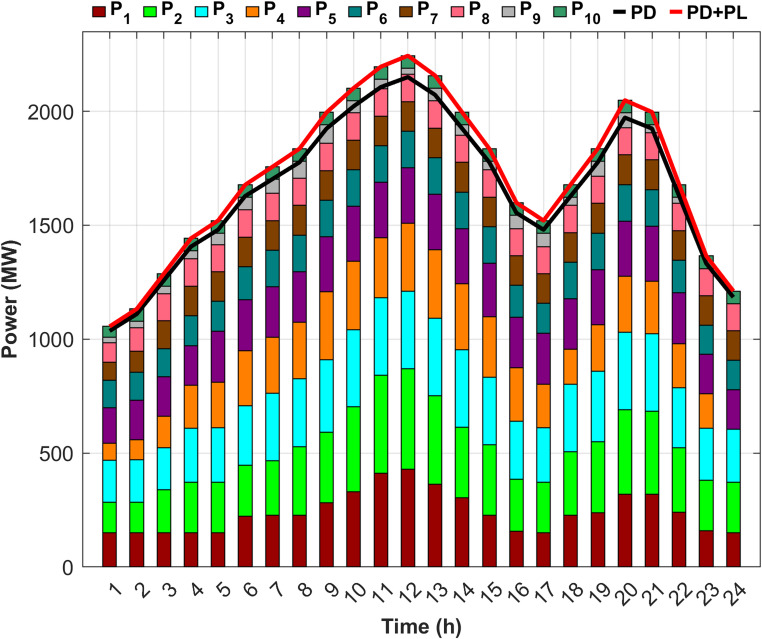
Detailed power generation and demand per hour for Scenario 2.

Figure 7 provides a graphical representation of the optimal power generated by all units to meet the demand load and system losses.

Figure 7 demonstrates the ability of the proposed DGC technique to extract the optimal capacity from each unit and maintain power balance throughout the day, despite unit 9 being out of service for the last two hours and unit 4 for the last hour. However, the proposed technique effectively compensated for the absence of the two units by redistributing power to the remaining units in an optimal manner, ensuring the lowest generation cost. Figure 7 also indicates an increase in the generation of the first five units compared to Scenario 1, with their contribution rising to 71.5% of the required load to offset the capacity shortfall caused by the fuel shortage.

Table 11 in Appendix A presents the detailed generated power, including the transmission losses for each time step, obtained using the MPA for Scenario 2.

Table 11 verifies that all generating units operate within the designated generation limits and satisfy the power balance requirements. Furthermore, the results demonstrate adherence to both minimum and maximum ramp rate constraints, excluding generator shutdown conditions. The highest recorded variations were 80 MW, 80 MW, 50 MW, 30 MW, and 30 MW for units 1, 2, 4, 7, and 9, respectively.

At 24:00, unit 4 experienced a sudden outage while operating at a capacity of 152.36 MW. The abrupt shutdown resulted in a complete power drop of 152.36 MW, exceeding the 50 MW ramp rate constraint. To mitigate these risks, several strategies could be proposed, including:

- Implementing gradual load reduction.
- Employing ramp-aware dispatch scheduling^43,44^.
- Adopting a smart energy management strategy to predict the violation of constraints, such as ramp rate, and thus advises the best routines, opening the window for future research.

#### Scenario 3: DEED with RES and fuel shortages in Units 4 and 9 without DGC

**Fig. 8:**
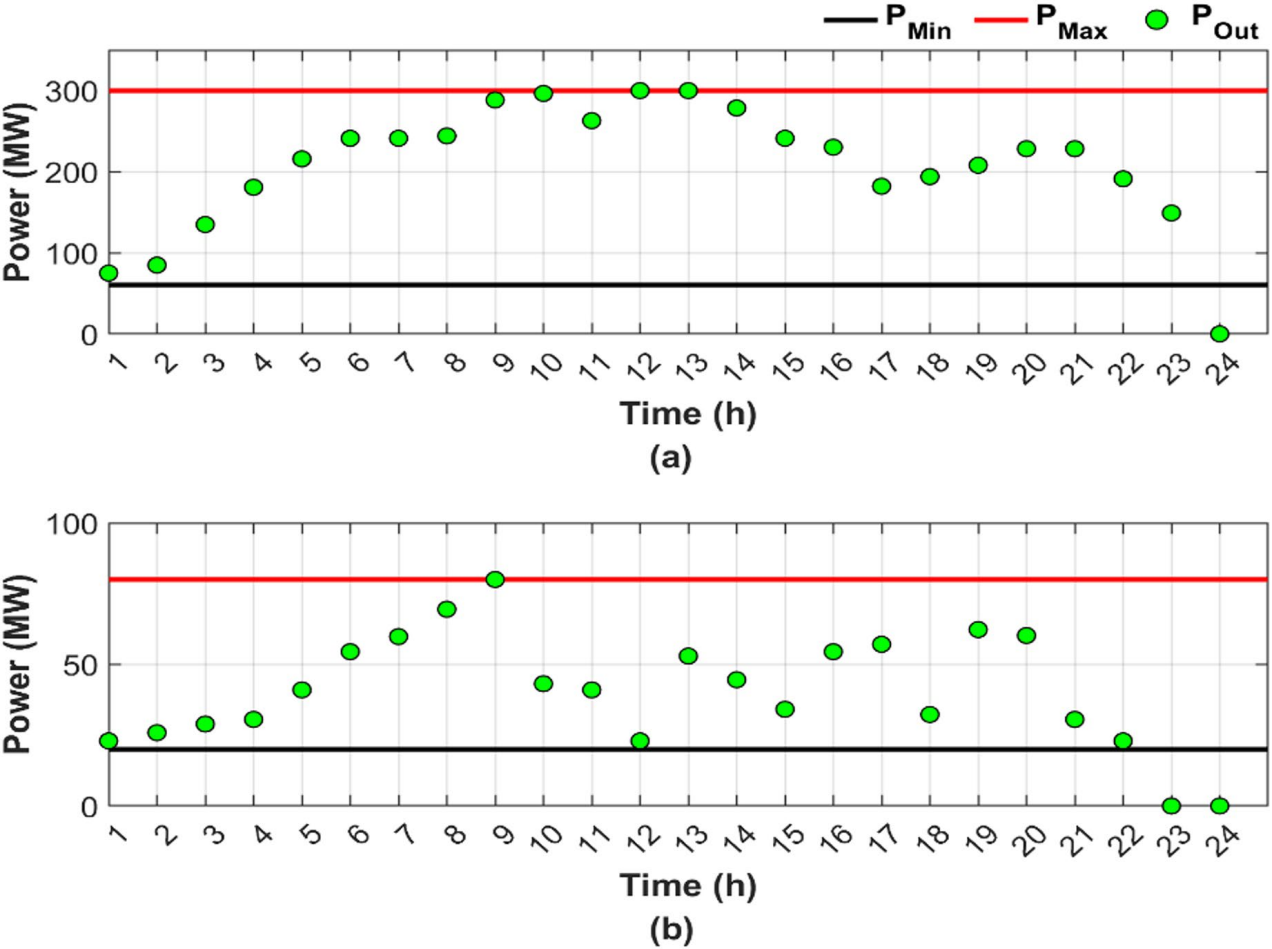
Hourly optimal power generation for Scenario 3: (**a**) unit 4 output and (**b**) unit 9 output (red line: maximum power generation limit, black line: minimum power generation limit).

In this scenario, the traditional DEED with fuel shortage conditions, including two wind stations and two PV stations, is adopted to evaluate the capability of RES to offset the power deficit caused by fuel shortages. DGC is not employed in the current scenario. Figure 8 shows the optimal output power for units 4 and 9 under fuel shortage conditions, considering PV and wind generation, with Fig. 8a corresponding to unit 4 and Fig. 8b to unit 9.

Figure 8 illustrates the fixed generation limits, highlighting the potential impact on supply security and economic operation of the power system, particularly under fuel shortage operating conditions. In practice, fuel shortages impose additional constraints, limiting the available capacity at each hour to a maximum value determined by the availability of fuel. If the optimal generation level falls below the hourly maximum value, the optimization proceeds successfully, and capacity is allocated accordingly. Conversely, if the optimal value exceeds the maximum power permitted by the available fuel, the generator is constrained to produce only up to the fuel-limited maximum.

Figure 8 also shows that due to fuel shortages, the output capacities of the affected units are expected to decrease relative to scenarios with sufficient fuel, as in Scenario 1. Nevertheless, even with the integration of RES, units 4 and 9 were unable to utilize the available fuel in an optimal manner, fully contributing 12.19% and 2.37% of the total load, respectively.

**Fig. 9:**
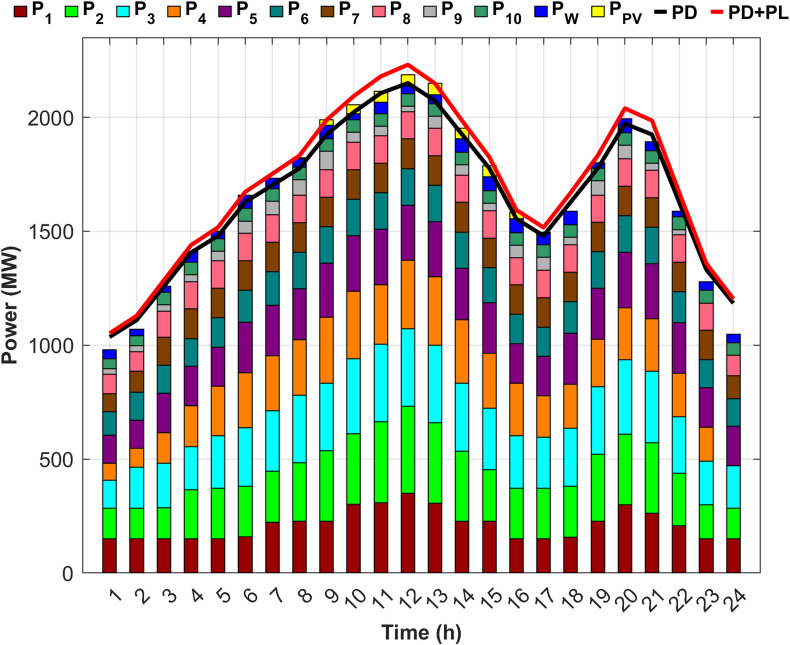
Detailed power generation and demand per hour for Scenario 3.

Figure 9 provides a graphical illustration of wind and PV power generation, along with the corresponding optimal power output for each generating unit.

Scenario 3 aims to optimize generation levels for units experiencing fuel shortages within an unrealistic fixed-limit framework. Consequently, compensating for any unaddressed shortfall from the proposed units depends on the output of RES. However, Fig. 9 illustrates that, despite the integration of RES, the output of the RES was insufficient to cover the shortage throughout most of the day, contributing only 3.37% toward an estimated 6% power deficit. Therefore, even after incorporating RES, a gap of approximately 2.53% in the total required load remains.

Table 12 in Appendix A presents the detailed generated power, including the transmission losses for each time period obtained using the MPA for Scenario 3.

Table 12 reveals that the generating units were unable to fulfill the required load throughout the day, despite the integration of RES, leading to varying levels of deficit throughout the day. The highest observed power variations were 80 MW, 80 MW, 50 MW, 30 MW, and 30 MW for units 1, 2, 4, 7, and 8, respectively. All the power variations remained within the allowable ramp rate limits, except for unit 9. Between 9:00 and 10:00, unit 9 experienced a decrease of 36.82 MW, exceeding the permissible ramp-down limit. Again, the strategies proposed for exceeding ramp rate and other limits in Scenario 2 could be adopted for unit 4 in the time zone of 23:00 and 24:00.

#### Scenario 4: DEED with RES and DGC for fuel shortages in Units 4 and 9

The DEED with DGC under fuel shortage conditions, incorporating two wind stations and two PV stations, is investigated to study the effectiveness of DGC in compensating for the power deficit and achieving 100% power security, while optimizing total generation costs.

To emphasize the impact of the DGC technique on modifying generation bounds in response to fuel availability, Table 8 presents the hourly updated generation limits for the targeted units under this scenario. This table is provided independently to demonstrate the effectiveness of the DGC approach and to facilitate precise interpretation of the results.

**Table 8:** Hourly updated generation limits for units 4 and 9 under Scenario 4.

Hour	*P*_4, min_ (MW)	*P*_4, max_ (MW)	*P*_9, min_ (MW)	*P*_9, max_ (MW)	Hour	*P*_4, min_ (MW)	*P*_4, max_ (MW)	*P*_9, min_ (MW)	*P*_9, max_ (MW)
1	60	75.02966	20	22.97344	13	60	300	20	70.61139
2	60	88.79205	20	27.04249	14	60	288.3282	20	44.93365
3	60	179.0031	20	31.80463	15	60	265.6557	20	37.80267
4	60	228.7905	20	35.08282	16	60	257.0766	20	57.99293
5	60	258.0739	20	50.31224	17	60	259.1286	20	59.11289
6	60	261.499	20	55.08966	18	60	228.8393	20	35.59182
7	60	268.9175	20	60.94274	19	60	210.8934	20	75.57015
8	60	297.2698	20	73.97977	20	60	234.4484	20	65.03965
9	60	300	20	80	21	60	230.4629	20	35.30266
10	60	300	20	53.28519	22	60	193.468	20	26.91741
11	60	263.7698	20	41.91485	23	60	152.3262	0	0
12	60	300	20	26.27066	24	0	0	0	0

Table 8 also illustrates the hourly variation of the generation limits due to changes in available fuel quantities. When comparing with Table 7 for Scenario 2, it is observed that for unit 4, the maximum generation limit increases relative to Scenario 2. This behavior is attributed to the integration of RES, which covers a portion of the load, particularly during hours 16–18, where load levels are relatively low and RES output is high. In contrast, unit 9 exhibits nearly identical generation limits to those in Scenario 2. This is because unit 9 has a relatively low generation capacity, limiting its flexibility across all scenarios. Conversely, unit 4, with a larger generation capacity, allows a wider search space for optimal output, making its generation limits more sensitive to changes in fuel availability and the contribution of RES.

**Fig. 10:**
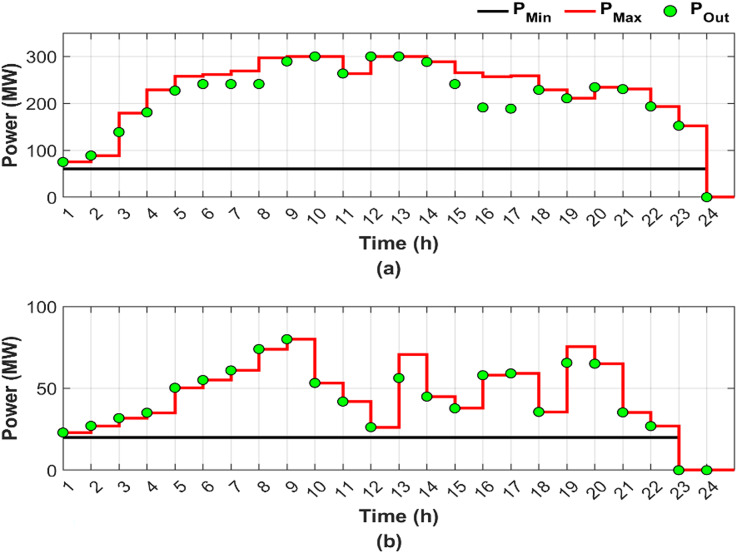
Hourly optimal power generation for Scenario 4: (**a**) unit 4 output and (**b**) unit 9 output (red line: maximum power generation limit, black line: minimum power generation limit).

Figure 10 provides a detailed illustration of the optimal output power profiles of units 4 and 9 under fuel shortage conditions, taking into account PV and wind generation, with two subfigures, including Fig. 10a corresponding to unit 4 and Fig. 10b corresponding to unit 9.

A comparison between Figs. 6 and 10 demonstrates that the integration of RES has expanded the search boundaries during certain hours, particularly between 6:00 and 18:00, due to the resulting reduction in fuel consumption. Expanded search boundaries increased flexibility, enhancing the search process during the periods of RES availability. However, the system operates similarly to Scenario 2 without RES integration outside the time zone 6:00 and 18:00. The effectiveness of the DGC method is reflected in the adaptability to both fuel shortage conditions and the incorporation of RES. Due to the fuel shortage, a reduction in the output capacities of the two targeted units is expected compared to the capacities in Scenario 1 under sufficient fuel availability. However, even with the integration of RES, units 4 and 9 optimally utilized all the fuel available, contributing 12.35% and 2.54% of the total load, respectively.

**Fig. 11:**
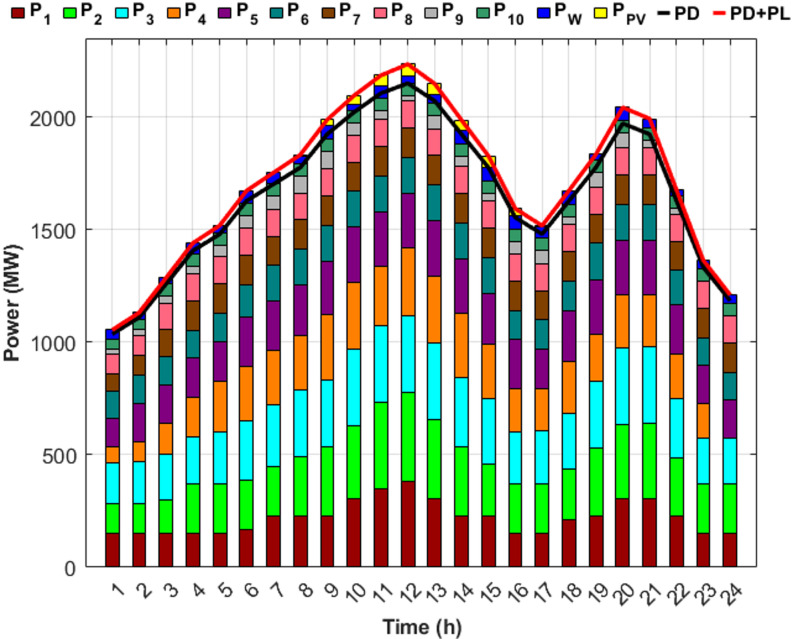
Detailed power generation and demand per hour for Scenario 4.

Figure 11 provides a graphical representation of the wind and PV power generation alongside the optimal power output for each generating unit.

Figure 11 shows that DEED with DGC under fuel shortage and the inclusion of RES effectively fulfills the load throughout the day, achieving 100% supply security. Scenario 3 failed to attain full power security when the DEED was used for the same operating conditions, but without incorporating DGC. Figure 11 also shows that the inclusion of wind and PV power reduced the generation power on the remaining units during the hours of RES availability, thereby facilitating power balance across the entire day. The generation share of the first five units is 68.28% of the required load, following the integration of RES, contributing 3.37% of the total load.

Table 13 in Appendix A presents the detailed generated power, including the transmission losses for each time period, obtained using the MPA for Scenario 4.

Table 13 confirms that all generating units operate within the specified generation limits and fulfill the power balance constraints. Additionally, the results indicate full compliance with both the minimum and maximum ramp rate limits, excluding generator shutdown conditions. The maximum observed variations are 80 MW, 50 MW, 30 MW, 30 MW, and 30 MW for units 2, 4, 7, 8, and 9, respectively. Again, the strategies proposed for exceeding ramp rate and other limits in Scenario 2 could be adopted for unit 4 in the time zone of 23:00 and 24:00.

### Power security against fuel cost and emissions

The results are illustrated in Figs. 6, 7, 8, 9, 10 and 11 demonstrate the efficiency of the proposed DEED approach with DGC in fulfilling load demand under limited fuel conditions. The proposed technique ensures supply continuity and achieves total system security. Regardless of fuel availability, the integration of wind and PV power generation helps the proposed DEED with DGC to consistently and optimally manage the system, thereby minimizing generation costs. Table 9 presents a comparison of total fuel cost, emissions, and power security over 24 h across the four considered scenarios.

**Table 9:** Comparison of total fuel cost, emissions, and supply security.

Scenario	Fuel cost ($)	Emissions (lb)	Power security (%)
Scenario 1 (Full fuel)	2.51E+06	3.05E+05	100
Scenario 2 (limited fuel with DGC)	2.57E+06	3.25E+05	100
Scenario 3 (RES without DGC)	2.33E+06	2.68E+05	97.4
Scenario 4 (RES with DGC)	2.42E+06	2.87E+05	100

Table 9 indicates that the RES without DGC case, Scenario 3, yields the lowest fuel cost and the lowest emission levels, achieving a 3.72% reduction in fuel cost and a 6.62% reduction in emissions compared to Scenario 4. However, Scenario 3 only supplied approximately 97.4% of the required load, thereby failing to ensure power security. Power deficit is the primary reason behind the reduced generation costs and emissions. Meanwhile, Table 9 shows that the RES with DGC in Scenario 4 successfully met the required load, adhered to all imposed constraints, and achieved the lowest possible cost and emission levels among the viable solutions.

Table 9 reveals that, under fuel shortage conditions, the DEED approach incorporating DGC and RES achieves the lowest fuel cost and emissions compared to the other evaluated scenarios. The elevated fuel costs and emissions in Scenario 2 stem from the necessity to identify the optimal generation capacity for each unit within the operational limits, driven by fuel shortages. Fuel shortages may affect types that typically offer lower costs and emissions. Consequently, maintaining power system security requires compensating for power deficits using alternative fuel types, regardless of the higher costs or emission levels. Table 9 also indicates that the DEED approach incorporating DGC and RES achieved a reduction in fuel costs of 3.59% compared to the full fuel case, Scenario 1, and 5.84% compared to the DGC scenario, while also lowering emissions by 5.9% and 11.69% relative to the first and second scenarios, respectively.

Table 9 indicates that adopting DGC results in an increase in the emission level, as evidenced by comparisons between the counterpart scenarios: Scenarios 1 and 2, and Scenarios 3 and 4. However, DGC also resulted in 100% security, while the system is suffering from a fuel shortage. Thus, a compromise has to be made between supply security and emissions.

In summary, the analysis highlights the following key findings. RES provides additional capacity to the system, thereby mitigating the impact of fuel shortages. In certain cases, RES capacity fully compensates for the deficit, whereas in other cases, only partial compensation occurs, depending on the available RES capacity. Notably, the contribution of RES occurs without requiring intervention from conventional generating units, as shown in Scenario 3. In contrast, the DGC method functions as a central controller, strategically coordinating all available units to address the capacity shortfall. The DGC directs units with surplus fuel to increase generation output, thereby improving power security, all within an optimization framework aimed at minimizing fuel costs, as in Scenarios 4.

## Conclusion

A DEED approach incorporating DGC was applied to optimize the utilization of the available fuel. Firstly, the promising optimizer, MPA, is identified via performance and statistical comparison between MPA, WaOA, and PSO. The results indicated that MPA delivered the most favorable performance compared to WaOA and PSO. For instance, for a 10-unit system under the 1000 MW load scenario, MPA and WaOA achieved reductions in generation cost of 0.120% and 0.119%, respectively, compared to PSO. Moreover, MPA achieved a decrease in standard deviation of 80.18% and 99.82% compared to WaOA and PSO, respectively. Then, MPA is used to analyze the performance of a 10-unit system under different operating conditions, including limited fuel, DGC, and RES power. The results highlight that integrating RES power with the DEED approach incorporating DGC enables effective system control under various fuel-limited conditions, ensuring operational requirements are met while avoiding the production of non-real power.

Relying only on RES failed to meet the required load because fuel shortages often occur suddenly and unpredictably, resulting in unanticipated capacity constraints. The DEED strategy, which combines DGC and RES, achieved fuel cost reductions of 3.59% and 5.84% compared to the normal operation and fuel shortage scenarios without RES, respectively, while also decreasing emissions by 5.9% and 11.96% relative to the previous scenarios. The following conclusions could be summarized:

- The literature on the DEED problem inadequately addresses fuel shortages in specific types and/or various types, thereby restricting the reliability of the findings.
- Integrating RES with DEED in all previous references aimed to reduce costs and emissions, rather than compensate for the capacity loss resulting from fuel shortages.
- The adaptability and efficiency of the DGC technique can be attributed to the incorporation of flexible limits, allowing generation boundaries to adjust based on the available fuel quantity. The proposed DGC technique also enhances supply security and helps reduce operating costs.
- The results presented in the study demonstrate that MPA outperformed WaOA and PSO in terms of generation cost, standard deviation, confidence interval, and various other statistical measures.

The proposed DGC ensures flexible power limits even under extreme operating conditions involving the loss of fuel from one or more units. The DGC maintains generation until the zero fuel level is reached. However, some operational constraints could be violated under such a strategy, for example, ramp rate limits. The tendency would usually be to adhere to the generation limit restrictions. The damage resulting from not adhering to the ramp rate limits would depend on the resulting reduction or increase in capacity and the tolerance available to the generating unit, as specified by the manufacturing specifications. Future research will focus on implementing effective solutions to the problem of exceeding ramp rate limits, ensuring the safe and stable operation of generating units by formulating a DEED model incorporating flexible scheduling for capacity allocation among units. The approach aims to prevent any unit from operating at high capacity before anticipated failures, allowing for a gradual reduction in the generator’s output, avoiding abrupt decreases in generation. Therefore, future strategies involving smart energy management algorithms and backup fuel supply should be considered, with further examination required. Further studies are needed to quantify the permissible levels of RES penetration in ED and stability assessments. Future work will also focus on incorporating the stochastic variations of RES for real-world systems to capture the inherent uncertainty more accurately and to comprehensively evaluate the impact of such variability on overall system reliability.

## Data Availability

All data generated or analyzed during this study are included in this published article.

## Appendix A

The detailed results of the MPA for the different operating scenarios are presented in Tables 10, 11, 12 and 13. The tables are included to act as a tool for verifying the results and confirming applicability. Moreover, the tables could guide researchers in the proposed field and enable cross-comparisons for their work.

**Table 10:** Dispatch results of the MPA for Scenario 1.

Hour	*P*_1_ (MW)	*P*_2_ (MW)	*P*_3_ (MW)	*P*_4_ (MW)	*P*_5_ (MW)	*P*_6_ (MW)	*P*_7_ (MW)	*P*_8_ (MW)	*P*_9_ (MW)	*P*_10_ (MW)	*P*_L_ (MW)
1	150	135	153.26	120.42	122.87	113.2	80	85.31	52.06	43.44	19.56
2	150	135	185.19	127.71	123.29	122.4	93.06	85.31	55.46	55	22.42
3	150	135	185.33	177.71	172.73	122.45	93.08	115.31	80	55	28.61
4	150	215	219.59	180.95	172.74	125.99	123.08	120	80	55	36.35
5	150.01	222.27	228.89	184.85	221.04	128.65	129.59	120	80	54.99	40.29
6	217.8	222.27	255.54	234.85	222.59	139.69	129.59	120	80	54.99	49.32
7	226.62	223.31	297.19	241.25	222.6	160	129.98	120	80	54.99	53.94
8	226.63	299.34	297.4	244.65	222.63	160	130	120	80	55	59.65
9	284.57	309.53	318.13	294.65	243	160	130	120	80	55	70.88
10	313.09	360.44	340	300	243	160	130	120	80	55	79.53
11	371.66	394.2	340	300	243	160	130	120	80	55	87.86
12	399.24	415.22	340	300	243	160	130	120	80	55	92.46
13	348.37	380.05	340	300	243	160	130	120	80	55	84.42
14	281.48	309.53	315.82	300	243	160	130	120	80	55	70.83
15	226.63	293.95	297.38	250	222.61	160	130	120	80	55	59.57
16	150.8	222.26	244.86	239.39	222.6	133.85	129.59	120	80	55	44.35
17	150	222.27	232.14	229.6	172.77	128.91	129.59	120	80	54.99	40.27
18	214.76	222.27	253.54	241.24	222.6	138.29	129.59	120	80	55	49.29
19	226.63	299.9	297.4	244.13	222.6	160	130	120	80	55	59.66
20	303.25	321.58	340	294.13	243	160	130	120	80	55	74.96
21	281.38	309.54	315.91	300	243	160	130	120	80	55	70.83
22	205.94	229.53	248.77	250	222.6	135.84	129.59	120	80	55	49.27
23	150	149.53	185.19	200	172.73	122.45	129.22	119.97	80	55	32.09
24	150	135	184.97	170.09	122.89	122.42	99.21	89.97	80	54.88	25.43

**Table 11:** Dispatch results of the MPA for Scenario 2.

Hour	*P*_1_ (MW)	*P*_2_ (MW)	*P*_3_ (MW)	*P*_4_ (MW)	*P*_5_ (MW)	*P*_6_ (MW)	*P*_7_ (MW)	*P*_8_ (MW)	*P*_9_ (MW)	*P*_10_ (MW)	*P*_L_ (MW)
1	150	135	183.75	75.03	155.33	120.45	80	85.31	22.98	47.88	19.73
2	150	135	185.39	88.79	172.73	122.45	93.06	103.04	27.04	55	22.5
3	150	187.93	185.2	138.79	172.73	122.45	123.06	120	31.81	55	28.97
4	150.15	222.26	237.45	188.77	173.17	130.9	129.59	120	35.08	55	36.37
5	150	222.27	238.38	201.2	222.59	130.9	129.59	120	50.31	55	40.24
6	223.3	222.27	262.79	241.24	222.6	145.45	129.59	120	55.09	55	49.33
7	226.62	238.57	297.4	244.94	222.61	160	130	120	60.94	55	54.08
8	226.62	302.01	297.4	248.05	222.62	160	130	120	73.98	55	59.68
9	282.54	309.53	316.73	298.05	243	160	130	120	80	55	70.85
10	331.23	370.99	340	298.51	243	160	130	120	53.29	55	80.02
11	411.23	430.69	340	263.65	243	160	130	120	41.91	55	89.48
12	428.47	440.92	340	300	243	160	130	120	26.27	55	93.66
13	363.73	388.87	340	300	243	160	130	120	56.27	55	84.87
14	303.25	310.83	339.94	288.31	243	160	130	120	44.93	55	71.26
15	226.64	308.9	297.4	265.43	234.58	160	130	120	37.8	55	59.75
16	156.43	228.9	254.53	234.48	222.6	138.9	129.59	120	57.99	55	44.42
17	150.1	222.27	238.16	192.15	222.51	131.37	129.59	120	59.11	54.99	40.25
18	226.62	278.32	297.39	152.75	222.62	160	129.99	120	35.59	55	50.28
19	239.06	309.53	311.15	202.74	243	160	130	120	65.59	55	60.07
20	319.02	371.19	340	244.85	243	160	130	120	65.04	55	76.1
21	319.47	364.21	340	229.5	243	160	130	120	35.3	55	72.48
22	239.47	284.21	262.95	193.64	222.6	143.98	129.59	120	26.92	54.99	50.35
23	159.47	222.27	226.15	152.36	172.75	127.29	129.59	120	0	55	32.88
24	150.01	222.27	232.01	0	172.76	129.45	129.59	120	0	55	27.09

**Table 12:** Dispatch results of the MPA for Scenario 3.

Hour	*P*_pv_ (MW)	*P*_w_ (MW)	*P*_1_ (MW)	*P*_2_ (MW)	*P*_3_ (MW)	*P*_4_ (MW)	*P*_5_ (MW)	*P*_6_ (MW)	*P*_7_ (MW)	*P*_8_ (MW)	*P*_9_ (MW)	*P*_10_ (MW)	*P*_L_ (MW)
1	0	40.5	150	135	122.33	75.03	122.87	102.22	80	85.31	22.97	43.42	15.74
2	0	27.9	150	135	178.13	84.88	122.87	121.90	93.06	85.31	25.88	44.60	19.16
3	0	25.5	150	135.89	194.78	134.88	172.73	122.45	123.06	115.31	28.94	55	26.35
4	0	47.4	150	215.89	187.08	180.83	172.73	122.45	129.59	120	30.57	55	32.59
5	0	33	150.05	222.27	230.76	215.87	172.73	128.63	129.59	120	40.99	55	37.51
6	0	57.12	158.84	222.27	255.75	241.23	222.59	139.96	129.59	120	54.49	55	44.44
7	0	46.5	223.61	222.27	265.53	241.24	222.60	146.68	129.59	120	59.83	55	49.85
8	4.8904	35.4	226.62	256.23	297.40	244.23	222.68	160	130	120	69.49	55	55.80
9	23.326	60	226.62	308.92	297.40	288.44	238.42	160	130	120	80	55	64.14
10	40.668	26.4	301.52	309.53	329.33	296.54	243	160	130	120	43.18	55	70.70
11	48.056	51.9	308.00	354.99	340	262.97	243	160	130	120	40.99	55	73.34
12	49.768	33.6	351.01	380.68	340	300	243	160	130	120	22.97	55	80.60
13	48.762	39.6	307.15	351.88	340	300	243	160	130	120	52.97	55	76.36
14	45.248	60	227.15	307.18	297.41	278.63	226.01	160	130	120	44.59	55	60.38
15	49.326	60	226.37	227.18	269.64	241.24	222.60	152.22	129.59	120	34.15	55	49.43
16	30.214	60	150	222.27	230.20	230.26	172.80	128.90	129.59	120	54.49	55	38.89
17	3.551	51	150	222.27	224.35	182.11	172.73	126.81	129.59	120	57.11	55	36.26
18	0	60	157.69	222.27	255.06	193.96	222.60	138.87	129.59	120	32.30	55	40.66
19	0	24.6	226.62	294.07	297.39	208.01	222.61	160	129.98	120	62.30	55	55.98
20	0	60	299.06	309.53	327.69	228.39	243	160	130	120	60.21	55	67.06
21	0	39	262.18	309.53	314.63	228.39	243	160	130	120	30.57	55	61.39
22	0	25.8	207.13	229.53	248.50	191.28	222.60	135.77	129.59	120	22.97	55	43.03
23	0	37.8	150	149.55	191.33	149.08	172.73	122.46	129.59	120	0	55	26.70
24	0	37.8	150	135	185.19	0	172.73	122.44	99.59	90	0	55	18.65

**Table 13:** Dispatch results of the MPA for Scenario 4.

Hour	*P*_pv_ (MW)	*P*_w_ (MW)	*P*_1_ (MW)	*P*_2_ (MW)	*P*_3_ (MW)	*P*_4_ (MW)	*P*_5_ (MW)	*P*_6_ (MW)	*P*_7_ (MW)	*P*_8_ (MW)	*P*_9_ (MW)	*P*_10_ (MW)	*P*_L_ (MW)
1	0	40.5	150	135	177.47	75.03	122.87	120.45	80	85.31	22.97	44.65	18.25
2	0	27.9	150	135	182.61	88.79	172.46	122.41	93.06	85.31	27.04	46.81	21.39
3	0	25.5	150	146.83	204.03	138.79	172.73	122.49	123.06	115.31	31.81	55	27.55
4	0	47.4	150	222.27	204.53	180.89	172.73	122.5	129.59	120	35.08	55	33.99
5	0	33	150.01	222.27	229.48	227.3	172.77	128.75	129.59	120	50.31	55	38.48
6	0	57.12	167.27	222.27	260.26	241.24	222.6	142.95	129.59	120	55.09	55	45.39
7	0	46.5	226.61	222.27	273.04	241.24	222.6	155.2	129.6	120	60.94	55	60
8	4.8904	35.4	226.62	265.23	297.4	241.35	222.71	160	129.97	120	73.98	55	56.55
9	23.326	60	226.62	309	297.4	289.44	237.35	160	130	120	80	55	64.14
10	40.668	26.4	303.25	324.12	340	300	243	160	130	120	53.28	55	73.72
11	48.056	51.9	350.95	380.79	340	263.77	243	160	130	120	41.91	55	79.38
12	49.768	33.6	379.88	397.44	340	300	243	160	130	120	26.27	55	84.96
13	48.762	39.6	304.38	351.29	340	300	243	160	130	120	56.27	55	76.3
14	45.248	60	227.26	309.53	303.35	288.33	243	160	130	120	44.94	55	62.66
15	49.326	60	226.63	229.56	295.75	241.25	222.6	160	129.87	120	37.8	55	51.79
16	30.214	60	150	222.27	228.1	191.29	220.98	127.95	129.59	120	57.99	55	39.38
17	3.551	51	150.01	222.27	234.77	188.76	173.02	130.3	129.59	120	59.11	55	37.38
18	0	60	212.43	222.27	250.88	228.84	222.59	136.53	129.59	120	35.59	55	45.72
19	0	24.6	226.63	302.25	297.4	210.89	241.76	160	130	120	65.59	55	58.12
20	0	60	303.25	332.05	340	234.45	243	160	130	120	65.04	55	70.79
21	0	39	303.28	336.91	340	230.46	243	160	130	120	35.3	55	68.95
22	0	25.8	226.56	256.91	268.62	193.47	222.6	150.8	129.59	120	26.92	55	48.27
23	0	37.8	150	222.27	200.77	152.33	172.73	122.49	129.59	120	0	55	30.98
24	0	37.8	150	222.27	199.53	0	172.73	122.46	129.59	120	0	55	25.38
